# Potential of Insect Meals as Protein Sources for Meat-Type Ducks Based on In Vitro Digestibility

**DOI:** 10.3390/ani9040155

**Published:** 2019-04-09

**Authors:** Attawit Kovitvadhi, Pipatpong Chundang, Karun Thongprajukaew, Chanin Tirawattanawanich, Sunyanee Srikachar, Banthari Chotimanothum

**Affiliations:** 1 Department of Physiology, Faculty of Veterinary Medicine, Kasetsart University, Bangkok 10900, Thailand; pichandang@gmail.com; 2 Department of Applied Science, Faculty of Science, Prince of Songkla University, Songkhla 90110, Thailand; karun.t@psu.ac.th; 3 Innovation Cluster 2, Thailand Science Park, Ministry of Science and Technology, Pathum Thani 12120, Thailand; chanint07@gmail.com; 4 Department of Agriculture, Ministry of Agriculture and Cooperatives, Bangkok 10900, Thailand; sunyaneesrikachar@gmail.com; 5 The Queen Sirikit Department of Sericulture, Ministry of Agriculture and Cooperatives, Bangkok 10900, Thailand; tip3537@gmail.com

**Keywords:** cherry valley, cricket, crude protein, fruit fly, *Hermetia illucens*, house fly, locust, mealworm, silkworm

## Abstract

**Simple Summary:**

There has been a dramatic increase in duck meat consumption. As a result, ducks are an interesting alternative type of livestock. Animal-based proteins such as fishmeal and animal by-products are valuable nutrients with high digestibility, but they are associated with cost fluctuations, pathogen contamination, and environmental impacts. Therefore, plant-based proteins are used, but they have the disadvantages of inappropriate amino acid profiles, anti-nutritional factors, and mycotoxin contamination. Insect meal contains favorable nutrients and low production costs and is environmentally friendly; however, there is a large number of insect species. Therefore, the purpose of this investigation is to screen insects for their potential use as a protein source in the duck diet. Insect meal with a high proportion of low-digestible components was shown to have low digestibility. In conclusion, yellow mealworm larvae, giant mealworm larvae, lesser wax moth larvae, house fly larvae, mulberry silkworm pupae, and American cockroach nymph have the potential to be alternative protein sources for ducks.

**Abstract:**

There has been a dramatic increase in duck meat consumption. As a result, ducks are an interesting alternative type of livestock. Animal-based proteins such as fishmeal and animal by-products are valuable nutrients with high digestibility, but they are associated with cost fluctuations, pathogen contamination, and environmental impacts. Therefore, plant-based proteins are used, but they have the disadvantages of inappropriate amino acid profiles, anti-nutritional factors, and mycotoxin contamination. Insect meal contains favorable nutrients and low production costs and is environmentally friendly; however, there is a large number of insect species. Therefore, the purpose of this investigation is to screen insects for their potential use as a protein source in the duck diet. Insect meal with a high proportion of low-digestible components was shown to have low digestibility. Yellow mealworm larvae, giant mealworm larvae, lesser wax moth larvae, house fly larvae, mulberry silkworm pupae, and American cockroach nymph have the potential to be alternative protein sources for ducks. Insect meal has been widely studied and is used in animal nutrition to replace common protein sources that have several disadvantages and to promote sustainability in animal production. Two-step in vitro digestibility using crude enzyme extracts from digestive tracts of meat-type ducks (Cherry Valley) was performed on general protein sources and insect meals to compare the in vitro digestibility of organic matter (OMd) and crude protein (CPd). Variation in chemical components between different types of insect meal was found. A positive correlation was found between OMd and the ether extract composition in insect meal, whereas a negative correlation was shown between crude fiber and acid detergent fiber. Contrasting relationships were found between CPd and crude fiber and acid detergent fiber in insect meal. In conclusion, the yellow mealworm larvae (*Tenebrio molitor*), giant mealworm larvae (*Zophobas morio*), lesser wax moth larvae (*Achroia grisella*), house fly larvae (*Musca domestica*), mulberry silkworm pupae (*Bombyx mori*), and American cockroach nymph (*Periplaneta americana*) are potential protein sources for ducks based on OMd and CPd digestibility after screening with an in vitro digestibility technique.

## 1. Introduction

Duck production increased by 6.89% from 2014 to 2017 (1150 million heads), with the highest production yield reported in China (63%) followed by Vietnam, Bangladesh, Indonesia, the Russian Federation, Myanmar, France, India, and Thailand [1]. Duck production plays an important role in several Asian countries, not only for large commercial scale production but also for individuals in poverty. The advantages of ducks are their great adaptation to fluctuating environments, high disease resistance, a large variety of feeds, a higher selling price, and a symbiotic production system (duck-cum-rice or duck-cum-fish system); these factors promote their value as alternative, sustainable livestock [2]. A major proportion of production costs is feed, which accounts for around 60–80% of costs; this particularly consists of raw protein materials. Therefore, the reduction of feed cost by using alternative by-products or indigenous raw materials, especially protein-based sources, could be a solution to this problem.

Fishmeal is an ideal protein source for animals, as it contains an appropriate amino acid profile and has high digestibility. However, a decrease in the usage of fishmeal as a protein-based raw material has occurred because several disadvantages of its use were discovered, such as cost fluctuations, pathogen contamination, and environmental impacts [3]. Therefore, plant-based protein has replaced fishmeal in several formulations for avian species [3,4]. Soybean meals, rapeseed meal, sesame meal, leucaena leaf meal, and duck weed (*Lemna minor*) were introduced as raw materials for ducks [3,4]. Supplementation of synthetic amino acids (methionine and/or lysine) is necessary because plant-based raw materials are deficient in limiting amino acids, which is a great obstacle for organic farming [3]. Anti-nutritional factors are observed in plants, leading to deterioration of their productivity, digestibility, health, and products [3,4]. Moreover, contamination with multiple mycotoxins (aflatoxins, fumonisins, and deoxynivalenol) was reported in soybean products [5], which is a serious problem as ducks are highly susceptible to mycotoxins compared with other avian species [6]. Therefore, alternative protein sources that can solve these problems while also providing sustainability should be studied [7].

Insects have attracted interest as alternative protein sources for animal feed, especially in poultry nutrition [7,8,9,10,11], based on their nutrient quality and environmentally friendly production system [12,13,14,15]. A previous study reported an increase on carcass weight in Alabio ducks fed live black soldier fly larvae as a supplement [16], and black soldier fly defatted larva meal was successfully used in Muscovy ducks [17]. However, the chitin contained in insect species may decrease the protein digestibility by physically protecting the protein from enzyme hydrolysis [18]. There are several potential insect species that could be used in this way in tropical countries [12]. A screening technique should be used before performing in an experiment on animals.

The in vitro digestibility technique not only has potential as a promising tool for estimating the suitability of protein sources for animals, but this technique probably provides a new way of measuring gastrointestinal functionality that can be used in multidisciplinary approaches to increase animal health, welfare, and performance, since the digestion of feed is the main function of the gastrointestinal tract system [18,19,20]. Generally, commercial enzymes from swine are used to measure in vitro digestibility [19,20]. However, pre-caecal amino acid digestibility differs between broilers, turkeys, and ducks [21]. Therefore, this study was designed to compare the in vitro digestibility of organic matter (OMd) and crude protein (CPd) from general protein sources and insect meal by using crude enzyme extract (CTX) from duck’s digestive organs.

## 2. Materials and Methods

Twenty digestive tracts from healthy commercial meat-type ducks (Cherry Valley) were collected at a commercial slaughterhouse during the evisceration process (Duck King Co., Ltd., Bangkok, Thailand). The digestive organs were immediately kept under ice and transported to the laboratory. The gastric mucosa, pancreas, and duodenal mucosa were separated, pooled, and homogenized with phosphate buffer solution (pH 7, 1:5 w/v). The homogenates were centrifuged at 18,000× *g* under 4 °C for 30 min to obtain the supernatant, which was stored at −80 °C to be used as the CTX of each organ. This study was carried out following the standard guidelines after being approved by the Institutional Animal Care and Use Committee of Kasetsart University, Bangkok, Thailand (ACKU61-VET-035).

High protein fishmeal (FMh), low protein fishmeal (FMl), chicken by-product meal (CBM), pork by-product meal (PBM), dehulled-soybean meal (DSB), and hulled-soybean meal (HSB), obtained from Bangkok Animal Research Center Co., Ltd., (Bangkok, Thailand) and Protector Nutrition Co., Ltd. (Bangkok, Thailand), were used as general protein sources. Seventeen types of insect meal were collected from local companies in Thailand (Dang Insect Distributor Co., Ltd., Pathumthani; Jae Tick Co., Ltd., Bangkok; Jerry Pet Supplies Co., Ltd., Bangkok; Jing Hleed Co., Ltd., Bangkok; Orgafeed Co., Ltd., Bangkok) and the government sector (Department of Agriculture and the Queen Sirikit Department of Sericulture of Ministry of Agriculture and Cooperatives, Bangkok, Thailand): the American cockroach (*Periplaneta americana*: PA, nymph), water scavenger beetle (*Hydrous cavistanum*: HC, adult), yellow mealworm (*Tenebrio molitor*: TM, larvae), giant mealworm (*Zophobas morio*: ZM, larvae), oriental fruit fly (*Bactrocera dorsalis*: BD, larvae), black soldier fly (*Hermetia illucens*: HI, prepupae), house fly (*Musca domestica*: MD, larvae), lesser wax moth (*Achroia grisella*: AG, larvae), mulberry silkworm (*Bombyx mori*: BMl, larvae), mulberry silkworm (*Bombyx mori*: BMp, pupae), eri silkworm (*Philosamia ricini*: PR, pupae), house cricket (*Acheta domesticus*: AD, adult), African mole cricket (*Gryllotalpa africana*: GA, adult), African cricket (*Gryllus bimaculatus*: GB, adult), ground cricket (*Gryllus testaceus*: GT, adult), oriental migratory locust (*Locusta migratoria*: LM, adult), and bombay locust (*Patanga succincta*: PS, adult). All insects were euthanized immediately by freezing at −20 °C for five days. After that, insect samples were dried at 60 °C for 48 h, grounded into particles of 1 mm in size, and preserved at −20 °C for use as substrates. All substrates were analyzed in triplicate to determine their dry matter (DM), crude ash, crude protein (CP), ether extract (EE) and crude fiber (CF) contents following the method from the Association of Official Analytical Chemists [22], whereas acid detergent fiber (ADF) was determined in insect meal [23]. 

A two-step in vitro digestibility test with CTX was performed in triplicate to simulate the conditions in the stomachs (first step) and small intestines (second step) of ducks. The procedures were adapted from previous research studies and followed the digestive physiology of ducks [18,19,20]. One hundred milligrams of each substrate were weighed for the OMd analysis, whereas the amount of protein of each substrate was calibrated at 100 mg protein (CP = %Nitrogen × 6.25) for the CPd analysis. The OMd analysis was performed at the same time with the same procedure, equipment, and researcher, whereas CPd was done in another set of experiments.

In the first step, prepared substrates were incubated in conical tubes with 5 mL of stimulated gastric fluid (SGF containing 0.0169 M NaCl, 0.0096 M KCl, and 0.0100 M HCl) at pH 2, 200 μL of gastric mucosal CTX, and 100 µL of 0.5% chloramphenicol. Each tube was covered with a screw cap and placed in an incubator shaker at 42 °C under constant shaking at 200 rpm for 4 h. In the second step, 10 mL of stimulated pancreas-intestinal fluid (SPIF; 0.0851 M NaCl, 0.0148 M KCl, 0.0300 M NaH_2_PO_4_, and 0.1700 M Na_2_HPO_4_) at pH 8 was added to stop the reaction of gastric enzymes and to prepare the optimal conditions for enzyme activity in the next step. Pancreatic and duodenal mucosal CTX were added in quantities of 100 μL each. Hydrolysis was continued for 8 h under the same conditions as in the first step. After hydrolysis, enzyme activity was terminated by adding 10% trichloroacetic acid. Undigested residues were collected by filtration using a filtered crucible (pore diameter: 40 to 100 µm), and they were washed with distilled water, 95% ethanol, and 96% acetone, respectively. The filtration procedure was performed in the cold extraction unit of the fiber analysis system (Fibertec system 1021 cold extractor, TecatorTM, Denmark). The DM of the residuals was recorded after drying overnight at 60 °C. The crude ash or CP content was determined in residuals by ignition at 600 °C or by using the Kjeldahl method [22] to obtain the information for the OMd and CPd calculations, respectively. OMd and CPd were calculated based on the difference between the input and output of OM or CP, respectively. The blank tubes (n = 6/experiment), which contained all reagents but without substrate, were placed in different positions in the incubator.

The statistical analysis was calculated using R-statistics with the Rcmdr Package in Rstudio. Pearson’s correlation was performed between the chemical compositions of insect meals and their percentages of OMd and CPd. A completely randomized design and one-way analysis of variance were used to evaluate the difference in OMd and CPd between general protein sources and insect meals (fixed factors) by using Tukey’s Honest Significant Difference for the post-hoc analysis. Hierarchical cluster analysis with Euclidean distance was used to establish a dendrogram of similarity between substrates for OMd and CPd. The statistical significance level was set at *p* < 0.05. 

## 3. Results

### 3.1. Chemical Composition of Substrates

Table 1 presented the chemical composition of general protein sources and types of insect meal. A large variation in chemical components in general protein sources and types of insect meal was found in this study. A high proportion of crude ash was observed in general protein sources from animals (FMh, FMl, CBM, and PBM) compared with plant-based sources (DSB and HSB) and insect meal. The EE percentage in DM in most of the types of insect meal was higher than that in animal-based proteins (9.60–14.8% DM), except for PR (11.5% DM), PS (12.9% DM), and LM (3.59% DM). A large variation in the CP content in animal-based protein (38.9–60.9% DM), plant-based protein (41.0–45.9% DM), and insect meal (37.6–64.5% DM) was presented. A low ADF proportion was identified in PA (5.53% DM), ZM (6.93% DM), BMp (7.63% DM), and TM (8.19% DM), whereas HC (20.6% DM) and GA (24.6% DM) contained high percentages of ADF. The ADF content of most types of insect meal was 10.1–15.8% DM.

### 3.2. Pearson Correlation between Chemical Composition and In Vitro Digestibility of Insect Meal

The results of the correlation analysis between the chemical composition and in vitro digestibility of insect meal are summarized in Table 2. A positive correlation between CF and ADF (r = 0.71; *p* < 0.01) was found, whereas a negative correlation was presented between CP and CF (r = −0.77; *p* < 0.01) for insect meal. A great relationship with statistical significance was observed between OMd and CPd (r = 0.89; *p* < 0.01). OMd was shown to be influenced by EE, CF, and ADF in insect meal. The higher proportion of EE in insect meal was correlated with the higher value of OMd (r = 0.77; *p* < 0.01), whereas CF (r = −0.56; *p* < 0.05) and ADF (r = −0.59; *p* < 0.05) were associated with lower OMd. The lower CPd percentage was affected by the higher proportion of CF and/or ADF in insect meal (r = −0.54 and −0.68; *p* < 0.05 and <0.01, respectively).

### 3.3. In Vitro Digestibility of Organic Matter and Crude Protein

**Figure 1:**
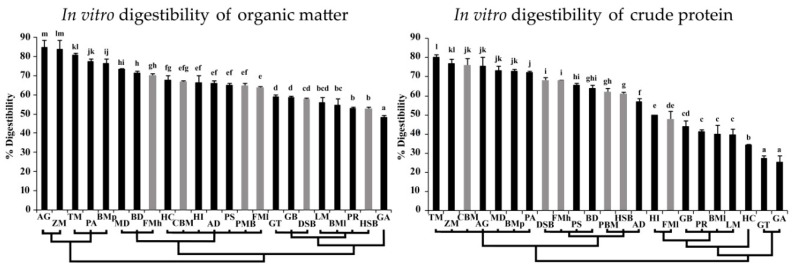
Dendrogram of the in vitro digestibility of organic matter and crude protein between general protein sources (grey column) and insect meal types (black column). The different superscripts on the top of column represent statistically significant differences at *p* < 0.05. Standard deviations are presented as error bars.

The comparing and grouping of OMd and CPd of general protein sources and types of insect meal is shown in Figure 1. The substrates were grouped into three major groups based on the results of the cluster analysis on OMd. The high OMd group contained only insect meal: AG, ZM, TM, PA and BMp. General protein-based raw materials (FMh, CBM, PBM and FMl) were included in the moderate OMd group with some of the insect meal types (MD, BD, HC, HI, AD and PS). GT, GB, LM, BMl, PR, and GA were clustered in the low OMd group with plant-based proteins (DSB and HSB). The results of the differences in CPd between the substrates are also summarized in Figure 1. 

General protein sources and insect meal for ducks based on CPd can be separated into five groups and arranged in ascending order as follows: Group 1 contains GT and GA; Group 2 contains only HC; Group 3 contains LM, BMl, PR, GB, FMl, and HI; Group 4 contains AD, HSB, PBM, BD, PS, FMh, and SMd; and Group 5 contains PA, BMp, MD, AG, CBM, ZM, and TM. The statistical differences in OMd and CPd between general protein sources and insect meal types are illustrated in Figure 1.

## 4. Discussion

The chemical compositions of types of insect meals in this study were in line with other experiments, showing a large variation between insect species and developmental stages [14,15,24,25]. The differences in mineral composition are likely to be the cause of the contrasting outcomes for ash contents between general protein sources from animals, plant-based sources, and insect meal [14,25].

Bones and intestinal organs are rich in mineral contents, which are present in fishmeal and animal by-products. Therefore, a higher amount of ash was observed in animal-based sources than in other sources.

The EE in insects was influenced by the developmental stage, season, sex, environment, insect diet, and postharvest methods [14,24,25,26,27]. A lower fat content is commonly observed in Orthoptera [14] which correlates with the results in this study for LM and PS. In contrast, other insect species showed high levels of EE contents in the order AD, GA, GB and GT because they were served commercial starter broiler diets with EE at 8%DM as their feed. The higher fat content in commercial diets promotes higher energy and lipid storage in the insect body compared to plant-based diets. Cassava leaves (*Manihot esculenta*) were used for the PR diet; therefore, they presented a low-fat content. Generally, insects in the larval stage contained a higher fat composition than those in the fully developed stage due to the energy preservation before metamorphosis, so the highest fat content was observed in AG larvae that were fed honey. 

The ADF content correlated with the chitin content in insects [18]. For this reason, ADF was used to estimate OMd and CPd in this study. The ADF proportion changed depending on the developmental stage of the insects, as those in the adult stage with an exoskeleton had higher ADF than those in the larval stage [8]. The CP content of insect meals exhibited a large variation, mainly due to diet [14,15,25]. Most of the insects in this study were produced in commercial farms and fed starter or finisher broiler diets, commercial insect diets, and/or organic waste, except for HC, LM, PS, and GA, which were harvested from the wild. Unfortunately, we do not have precise details of the diets for the insects in this study. Therefore, this hypothesis needs further study to be confirmed.

The CF content of insects could be used to estimate or replace the ADF because a statistically significant positive correlation was observed in this study. Moreover, CF may use to predict the chitin content of insects [18]. However, further study with a larger number of insect species should be done to confirm this hypothesis. OMd may be used to represent the CPd, as the OMd method is easier and less expensive to run than CPd and a great correlation was found between OMd and CPd in the current study. However, a difference between OMd and CPd for each insect species was presented, which reveals the inaccuracy of using this parameter. Therefore, it is still recommended that CPd is used to represent the CP digestibility efficiency. Based on the results of this current study, the EE, CF, and ADF contents in insect meal influenced the OMd. Fat can be easily digested by animal enzymes. Therefore, the EE content was significantly positively correlated with OMd. Raw materials with high proportions of CF and ADF have lower digestibility by animal enzymes. A negative correlation between fiber proportions (CF and ADF) and OMd was observed in this study, as well as the other research study [19]. 

Nitrogen molecules in samples are counted as crude proteins in the Kjeldahl method; thus, this method cannot differentiate between true protein and non-protein nitrogen compounds [22]. No correlation between CPd and CP contents in insect meal was found in this study. However, Marono et al. [18] found a positive correlation between the CP content in insect meal (HI) and CPd. The difference in in vitro digestibility techniques is likely to be the cause of the different outcomes. Moreover, the correlation in the study of Marono et al. [18] was calculated only in black soldier fly pre-pupae (HI). Therefore, CP is not suggested for use as a parameter to predict CPd in insect meal types other than HI. 

The amount of ADF and/or CF in insect meal is contrasted with CPd. ADF is a better predictor than CF based on the statistical results in this study. A negative correlation between these chemical compositions was also reported in another study [18]. The cause of this may be the poor digestion efficiency of animal enzymes for CF and ADF [16,18,21]. As the technique to evaluate the chitin content is complicated, ADF was suggested as a parameter to estimate chitin [28]. A larger variation in insect meal in term of species and chemical composition should be included to create the estimation equation in the further study.

AG, ZM, TM, PA, and BMp were higher in OMd than general protein sources and other types of insect meal. The high proportion of EE may be the cause of this, as described above. As the screening or selection of digestibility efficiency in raw protein materials is the objective of the study, CPd was suggested for use rather than OMd. However, the OMd of defatted substrates may correlate with the CPd. Therefore, further studies should confirm this hypothesis. MD, BD, HC, HI, AD, and PS were shown to be similar in OMd to general protein-based raw materials, whereas GT, GB, LM, BMl, PR, and GA were grouped with general plant-based raw materials. There has been no study about the screening of insect meal for ducks using in vitro digestibility techniques; however, a study in broilers, which compared OMd between cricket meal and general protein sources by using in vitro digestibility techniques, can be discussed. The OMd of AD was comparable to fishmeal, whereas GB and de-hulled soybean meal had lower values. The results in this study have similar trends to those observed in broilers [8,20]. However, a lower percentage of OMd was reported in broilers compared with this study, which was performed in ducks. The difference in enzyme activity between species [7] and the in vitro digestibility protocols used are likely to be the causes of the diverse consequences [21]. Therefore, the specific experimental design should be studied to compare the digestibility efficiency between species by using an in vitro technique.

GT, GA, HC, LM, BMl, PR, GB, FMl, and HI were low in CPd. Therefore, the usage of these insects for duck feed formulations should be considered in terms of the adverse effects of a low digestibility coefficient. Based on the CPd results in this study, AD, BD, and PS have the potential to replace FMh, DSB, PBM, and HSB. Moreover, PA, BMp, MD, AG, ZM, and TM have great potential to be alternative protein sources for ducks due to their high CPd values, which are similar to that of CBM. Marono et al. [18] reported higher CPd in HI at the pre-pupal stage than in TM using in vitro digestibility techniques, whereas TM had a higher CPd than the other insect meals in the current study. The differences in the chemical compositions of the insect meals and the use commercial enzyme from swine for the in vitro digestibility procedure are likely to be the causes of the contrasting outcomes. An improvement in the carcass percentage was observed in Alabio ducks fed with live black soldier fly larvae [16]. Based on the results from this study, the appropriate supplementation level is important and is related to the digestibility coefficient. Therefore, the use of a high percentage of HI in duck feed formulation should be avoided, as it has a low digestibility coefficient compared to general protein sources.

## 5. Conclusions

In conclusion, yellow mealworm larvae, giant mealworm larvae, lesser wax moth larvae, house fly larvae, mulberry silkworm pupae, and American cockroach nymph are suggested for use as alternative protein sources for ducks based on in vitro digestibility results. The use of these insects in the duck diet in different proportions should be studied to confirm the results of this study and to promote sustainable duck production.

## Figures and Tables

**Table 1:** Chemical composition of general protein sources and insect meals.

Substrates	Chemical Composition (%DM)					
	DM ^1^	Ash	CP	EE	CF	ADF
General protein sources (animal-based proteins)						
Fishmeal: high protein (FMh)	91.1	20.7	58.6	9.60	0.57	-
Fishmeal: low protein (FMl)	92.2	24.6	38.9	14.8	0.59	-
Chicken by-product meal (CBM)	95.5	16.3	60.9	10.2	2.46	-
Pork by-product meal (PBM)	95.3	33.4	46.2	10.4	0.33	-
General protein sources (plant-based proteins)						
Dehulled-soybean meal (DSB)	87.7	6.18	45.9	0	4.31	-
Hulled-soybean meal (HSB)	88.4	6.96	41.0	0	7.55	-
Insect meal						
Order: Blattodea						
*Periplaneta americana* (PA:nymph)	94.6	3.98	64.4	23.6	4.36	5.53
Order: Coleoptera						
*Hydrous cavistanum* (HC:adult)	86.3	1.88	41.9	38.3	14.7	20.6
*Tenebrio molitor* (TM:larvae)	97.1	5.95	53.0	31.0	8.47	8.19
*Zophobas morio* (ZM:larvae)	96.8	5.53	42.0	41.7	6.28	6.93
Order: Diptera						
*Bactrocera dorsalis* (BD:larvae)	95.1	9.41	45.2	31.3	5.94	13.6
*Hermetia illucens* (HI:prepupae)	91.8	9.54	37.9	30.1	12.3	11.2
*Musca domestica* (MD:larvae)	93.8	6.78	54.8	21.7	9.65	14.9
Order: Lepidoptera						
*Achroia grisella* (AG:larvae)	97.2	6.02	37.6	48.6	3.02	12.7
*Bombyx mori* (BMl:larvae)	96.7	10.1	61.2	17.6	5.39	13.6
*Bombyx mori* (BMp:pupae)	95.2	4.51	50.4	35.0	4.61	7.63
*Philosamia ricini* (PR:pupae)	92.6	7.15	64.5	11.5	8.53	10.1
Order: Orthoptera						
*Acheta domesticus* (AD:adult)	95.8	4.66	52.8	24.5	10.0	12.3
*Gryllotalpa africana* (GA:adult)	94.9	4.12	54.3	17.7	17.8	24.6
*Gryllus bimaculatus* (GB:adult)	92.2	5.05	53.3	22.6	8.98	13.5
*Gryllus testaceus* (GT:adult)	95.7	4.54	40.2	24.7	8.35	13.2
*Locusta migratoria* (LM:adult)	91.9	4.56	58.5	3.59	12.7	15.8
*Patanga succincta* (PS:adult)	92.0	4.29	63.3	12.9	15.1	12.8

DM = dry matter, CP = crude protein, EE = ether extract, CF = crude fiber, ADF = acid detergent fiber; ^1^ expressed as fresh matter.

**Table 2:** Correlation coefficients between chemical components of insect meals and in vitro digestibility of organic matter (OMd) and crude protein (CPd).

Parameters	CP	EE	CF	ADF	OMd	CPd
Ash	−0.05	−0.03	−0.35	−0.21	−0.05	0.11
CP		−0.77 **	0.11	−0.10	−0.39	−0.03
EE			−0.45	−0.23	0.77 **	0.47
CF				0.71 **	−0.56 *	−0.54 *
ADF					−0.59 *	−0.68 **
OMd						0.89 **

CP = crude protein, EE = ether extract, CF = crude fiber, ADF = acid detergent fiber; * correlation is significant at the 0.05 level (2-tailed); ** correlation is significant at the 0.01 level (2-tailed).

## References

[1] FAOSTAT. http://www.fao.org/faostat/en/#data.

[2] Adzitey F., Adzitey S.P. (2011). Duck production: Has a potential to reduce poverty among rural households in Asian communities—A review. J. World Poult. Res..

[3] Thongwittaya N. (2007). Substitution of plant protein for fish meal in the diet of laying ducks. Animal Sci. J..

[4] Fazhi X., Lvmu L., Jiaping X., Kun Q., Zhide Z., Zhangyi L. (2011). Effects of fermented rapeseed meal on growth performance and serum parameters in ducks. Asian Aust. J. Anim. Sci..

[5] Gutleb A.C., Caloni F., Giraud F., Cortinovis C., Pizzo F., Hoffmann L., Bohn T., Pasquali M. (2015). Detection of multiple mycotoxin occurrences in soy animal feed by traditional mycological identification combined with molecular species identification. Toxicol. Rep..

[6] Han X.Y., Huang Q.C., Li W.F., Jiang J.F., Xu Z.R. (2008). Changes in growth performance, digestive enzyme activities and nutrient digestibility of cherry valley ducks in response to aflatoxin B1 levels. Livest Sci..

[7] Biasato I., Gasco L., De Marco M., Renna M., Rotolo L., Dabbou S., Capucchio M.T., Biasibetti E., Tarantola M., Bianchi C. (2017). Effects of yellow mealworm larvae (*Tenebrio molitor*) inclusion in diets for female broiler chickens: implications for animal health and gut histology. Anim. Feed Sci. Technol..

[8] Bovera F., Piccolo G., Gasco L., Marono S., Loponte R., Vassalotti G., Mastellonea V., Lombardi P., Attia Y.A., Nizza A. (2015). Yellow mealworm larvae (*Tenebrio molitor*, L.) as possible alternative to soybean meal in broiler diets. Br. Poult Sci.

[9] Schiavone A., De Marco M., Martínez S., Dabbou S., Renna M., Madrid J., Hernandez F., Rotolo L., Costa P., Gai F. (2017). Nutritional value of a partially defatted and a highly defatted black soldier fly larvae (*Hermetia illucens* L.) meal for broiler chickens: Apparent nutrient digestibility, apparent metabolizable energy and apparent ileal amino acid digestibility. J. Anim. Sci. Biotechnol..

[10] Biasato I., Gasco L., De Marco M., Renna M., Rotolo L., Dabbou S., Capucchio M.T., Biasibetti E., Tarantola M., Sterpone L. (2018). Yellow mealworm larvae (*Tenebrio molitor*) inclusion in diets for male broiler chickens: Effects on growth performance, gut morphology, and histological findings. Poult. Sci..

[11] Dabbou S., Gai F., Biasato I., Capucchio M.T., Biasibetti E., Dezzutto D., Meneguz M., Plachà I., Gasco L., Schiavone A. (2018). Black soldier fly defatted meal as a dietary protein source for broiler chickens: Effects on growth performance, blood traits, gut morphology and histological features. J. Anim. Sci. Biotechnol..

[12] Hanboonsong Y., Jamjanya T., Durst B.P. (2013). Six-Legged Livestock: Edible Insect Farming, Collection and Marketing in Thailand.

[13] Van Huis A. (2013). Potential of insects as food and feed in assuring food security. Annu. Rev. Entomol..

[14] Makkar P.S.H., Tran G., Heuzé V., Ankersa P. (2014). State-of-the-art on use of insects as animal feed. Anim. Feed Sci. Technol..

[15] Sánchez-Muros M., Fernando G.B., Manzano-Agugliaro F. (2014). Insect meal as renewable source of food for animal feeding: A review. J. Clean Prod..

[16] Gunawan A., Erlina S., Samudera R., Syarif D.M., Noor M.Y., Lantu A.X. Effect of supplement maggot black soldier fly live on the percentage of carcass and weight of carcass of male Alabio ducks. Proceedings of the 1st International Conference on Food and Agriculture.

[17] Gariglio M., Dabbou S., Biasato I., Capucchio M.T., Colombino E., Hernandez F., Madrid Sanchez J., Martinez S., Gai F., Caimi C. Nutritional effects of the dietary inclusion of partially defatted *Hermetia illucens* larva meal in Muscovy duck. J. Anim. Sci. Biotechnol..

[18] Marono S., Piccolo G., Loponte R., Di Meo C., Attia Y.A., Nizza A., Bovera F. (2015). In vitro crude protein digestibility of *Tenebrio molitor* and *Hermetia illucens* insect meals and its correlation with chemical composition traits. Ital. J. Anim. Sci..

[19] Zhao F., Zhang L., Mi B.M., Zhang H.F., Hou S.S., Zhang Z.Y. (2014). Using a computer-controlled simulated digestion system to predict the energetic value of corn for ducks. Poult. Sci..

[20] Kovitvadhi A., Luapan J., Amarapitak P., Sriyaphai P., Buahom R., Cham-iam T., Chandang P., Leelehapongsathon K., Tirawattanawanich C., Thongprajukaew K. Screening three cricket species (*Gryllus bimaculatus*, *Acheta domestica* and *Modicogryllus confirmata*) for broiler diets by in vitro digestibility techniques. Proceedings of the 6th Mediterranean Poultry Summit.

[21] Kluth H., Rodehutscord M. (2006). Comparison of amino acid digestibility in broiler chickens, turkeys, and Pekin ducks. Poult. Sci..

[22] (2006). Association of Official Analytical Chemists, Official Method of Analysis.

[23] Van Soest P.J., Robertson J.B., Lewis B.A. (1991). Methods for dietary fiber, neutral detergent fibre, and nonstarch polysaccharides in relation to animal nutrition. J. Dairy Sci..

[24] Finke M.D. (2002). Complete nutrient composition of commercially raised invertebrates used as food for insectivores. Zoo Biol..

[25] Barroso F.G., Haro C., Sánchez-Muros M.J., Venegas E., Martínez-Sánchez A., Pérez-Bañón C. (2014). The potential of various insect species for use as food for fish. Aquacul.

[26] Adámková A., Mlček J., Kouřimská L., Borkovcová M., Bušina T., Adámek M., Bednářová M., Krajsa J. (2017). Nutritional potential of selected insect species reared on the Island of Sumatra. Int. J. Environ. Res. Public Health.

[27] Meneguz M., Schiavone A., Gai F., Dama A., Lussiana C., Renna M., Gasco L. (2018). Effect of rearing substrate on growth performance, waste reduction efficiency and chemical composition of black soldier fly (*Hermetia illucens*) larvae. J. Sci. Food Agric..

[28] Finke M.D. (2007). Estimate of chitin in raw whole insects. Zoo Biol..

